# Outcome of *TCF3-PBX1* positive pediatric acute lymphoblastic leukemia patients in Japan: a collaborative study of Japan Association of Childhood Leukemia Study (JACLS) and Children's Cancer and Leukemia Study Group (CCLSG)

**DOI:** 10.1002/cam4.221

**Published:** 2014-02-28

**Authors:** Daisuke Asai, Toshihiko Imamura, Yuka Yamashita, So-ichi Suenobu, Akiko Moriya-Saito, Daiichiro Hasegawa, Takao Deguchi, Yoshiko Hashii, Mikiya Endo, Naoki Hatakeyama, Hirohide Kawasaki, Hiroki Hori, Keizo Horibe, Keiko Yumura-Yagi, Junichi Hara, Arata Watanabe, Atsushi Kikuta, Megumi Oda, Atsushi Sato, for the Japan Association of Childhood Leukemia Study (JACLS) & Children's Cancer and Leukemia Study Group (CCLSG)

**Affiliations:** 1 Department of Pediatrics, Kyoto Prefectural University of Medicine, Graduate School of Medical ScienceKyoto, Japan; 2 Clinical Research Center, National Hospital Organization Nagoya Medical CenterNagoya, Japan; 3 Division of General Pediatrics and Emergency Medicine, Department of Pediatrics, Oita UniversityOita, Japan; 4 Department of Hematology/Oncology, Hyogo Prefectural Children's HospitalKobe, Japan; 5 Department of Pediatrics, Mie UniversityTsu, Japan; 6 Department of Pediatrics, Osaka UniversityOsaka, Japan; 7 Department of Pediatrics, Iwate Medical UniversityIwate, Japan; 8 Department of Pediatrics, Sapporo Medical University of MedicineHokkaido, Japan; 9 Department of Pediatrics, Kansai Medical UniversityHirakata, Japan; 10 Department of Pediatrics, Osaka General Medical CenterOsaka, Japan; 11 Department of Pediatrics, Osaka City General HospitalOsaka, Japan; 12 Department of Pediatrics, Nakadori General HospitalAkita, Japan; 13 Department of Pediatrics, Fukushima Medical SchoolFukushima, Japan; 14 Department of Pediatrics, Okayama UniversityOkayama, Japan; 15 Department of Pediatric Hematology/Oncology, Miyagi Children's HospitalSendai, Japan

**Keywords:** IKZF1 deletion, pediatric acute lymphoblastic leukemia, TCF3-PBX1

## Abstract

This study reviewed the clinical characteristics of 112 pediatric B-cell precursor acute lymphoblastic leukemia (BCP-ALL) patients with *TCF3-PBX1* fusion treated according to the Japan Association of Childhood Leukemia Study (JACLS) ALL02 protocol (*n* = 82) and Children's Cancer and Leukemia Study Group (CCLSG) ALL 2004 protocol (*n* = 30). The 3-year event-free survival (EFS) and overall survival (OS) rates were 85.4 ± 3.9% and 89.0 ± 3.5% in JACLS cohort, and the 5-year EFS and OS were 82.8 ± 7.0% and 86.3 ± 6.4% in CCLSG cohort, respectively, which are comparable to those reported in western countries. Conventional prognostic factors such as age at onset, initial white blood cell count, and National Cancer Institute risk have also no impact on OS in both cohorts. Surprisingly, the pattern of relapse in JACLS cohort, 9 of 82 patients, was unique: eight of nine patients relapsed during the maintenance phase and one patient had primary induction failure. However, bone marrow status and assessment of minimal residual disease on days 15 and 33 did not identify those patients. Interestingly, the two patients with *IKZF1* deletion eventually relapsed in JACLS cohort, as did one patient in CCLSG cohort. International collaborative study of larger cohort is warranted to clarify the impact of the *IKZF1* deletion on the poor outcome of *TCF3-PBX1* positive BCP-ALL.

## Introduction

The translocation t(1;19)(q23;p13) and its unbalanced variant der(19)t(1;19)(q23;p13) are well-known chromosomal abnormalities in pediatric B-cell precursor acute lymphoblastic leukemia (BCP-ALL) 1,2. This translocation results in the fusion of *TCF3* on 19p13 with *PBX1* on 1q23, generating the fusion gene *TCF3-PBX1* on derivative chromosome 19 3.

Although t(1;19)(q23;p13) was initially associated with poor prognosis in pediatric BCP-ALL, the recent development of intensified chemotherapy regimens has improved the outcome of this subgroup, resulting in a 5-year event-free survival (EFS) rate of ∼85−90% in western countries, which is similar to that of *TEL-AML1* positive or high hyperdiploid BCP-ALL 2,4–6. However, ∼10% of patients experience relapse with dismal prognosis 2,4, underscoring the importance of identifying reliable prognostic markers to improve the treatment of these patients. In the last decades, several studies have attempted to identify prognostic markers for this subgroup of pediatric BCP-ALL with unsatisfactory results 4,5,7. Classic prognostic factors, such as age at onset, initial white blood cell (WBC) count, National Cancer Institute (NCI) risk group, and type of chromosomal abnormality [balanced t(1;19) and unbalanced t(1;19)], did not have prognostic value in recent studies 4,5. Genetic analysis to identify alterations related to poor prognosis in pediatric BCP-ALL patients with *TCF3-PBX1* fusion has not been performed to date, with the exception of one study that analyzed the relationship between *TP53* mutation and poor prognosis in a small number of patients 8. Herein, we reviewed the clinical data of 112 pediatric BCP-ALL patients with *TCF3-PBX1* fusion, which is the largest such cohort reported to date. Additionally, we performed genetic analyses, including *IKZF1* and *TP53*, to determine the prognostic value of these genetic alterations in pediatric BCP-ALL with *TCF3-PBX1*.

## Design and Methods

### Patient cohorts and samples

From April 2002 to May 2008, 1138 patients aged 1–18 years with newly diagnosed BCP-ALL were enrolled in the Japan Association of Childhood Leukemia Study (JACLS) ALL02 study 9–11. The diagnosis of BCP-ALL was based on morphological findings of bone marrow (BM) aspirates and immuno-phenotypic analysis of leukemic cells by flow cytometry. Conventional cytogenetic analysis using G-banding was performed as part of the routine workup. Molecular studies using quantitative RT-PCR (RQ-PCR) for the detection of *TCF3-PBX1* were also performed as part of the routine workup (Table S1). Ph + ALL and infantile ALL patients were excluded from the study. Patients with Down syndrome were also excluded.

Bone marrow smears were examined under the microscope on days 15 and 33 (at the end of the induction phase) to evaluate the treatment response. M1, M2, and M3 marrow were defined as fewer than 5%, 5−25%, and more than 25% blast cells in the BM aspirate, respectively. Complete remission (CR) was defined as the absence of blast cells in the peripheral blood, fewer than 5% blast cells in the BM aspirate, normal cellularity and trilineage hematopoiesis, and absence of blast cells in the cerebrospinal fluid and elsewhere. RQ-PCR for *TCF3-PBX1* was also performed on days 15, 33, and 71 (at the end of consolidation) to determine minimal residual disease (MRD). The *GAPDH* gene was amplified as an internal control of RNA quality.

An independent validation cohort of 30 pediatric BCP-ALL patients with *TCF3-PBX1* fusion was enrolled from the Children's Cancer and Leukemia Study Group (CCLSG) ALL 2004 protocol between June 2004 and May 2009 12. The diagnosis of BCP-ALL was based on morphological and immuno-phenotypic analyses as described for the JACLS cohort. Patients with t(1;19)/der(19)t(1;19) determined by G-banding analysis or *TCF3-PBX1* fusion determined by RQ-PCR in the JACLS or CCLSG cohorts were enrolled in this analysis. Informed consent was obtained from the patients' guardians according to the Declaration of Helsinki; treatment and genetic study protocols were approved by the Institutional Review Boards of the participating institutions.

### Determination of *IKZF1* deletion by multiplex ligation-dependent probe amplification analysis

Genomic DNA was isolated from diagnostic BM or peripheral blood samples using the Qiagen DNeasy tissue and blood kit according to the manufacturer's instructions (Qiagen, Venio, the Netherlands). DNA specimens of 53 patients in the JACLS cohort and 22 patients in the CCLSG cohort were analyzed using the SALSA multiplex ligation-dependent probe amplification (MLPA) kit P335-A4 according to the manufacturer's instructions (MRC Holland, Amsterdam, the Netherlands) as described elsewhere 11,13.

### *JAK2* and *TP53* mutations analysis

To screen for the *JAK2* mutation in exons 16, 20, and 21 (accession number NM 004972), genomic DNA was extracted from diagnostic BM samples of two patients in the JACLS cohort harboring *IKZF1* deletions. Because the frequency of *JAK2* mutation has been reported to be quite rare and clustered in the patients with *IKZF1* deletion 11, we analyzed *JAK2* mutation in the patients with *IKZF1* deletion. To screen for *TP53* mutations in exons 5, 6, 7, 8, and 9 (accession number NM 000546), genomic DNA was also extracted from diagnostic BM samples of eight patients in the JACLS cohort who experienced relapse. Because *TP53* mutation was associated with relapsed *TCF3-PBX1* positive ALL 8, genomic DNA extracted from relapsed BM samples of four of the eight cases in the JACLS cohort was also used for *TP53* mutation screening. The primers used for *JAK2* mutation screening were as previously described 11. The primers used for *TP53* mutation screening are listed in Table S1. The PCR product was analyzed by direct sequencing using a BigDye Terminator sequencing kit (Applied Biosystems, Foster City, CA).

### Statistical analysis

Estimation of survival distributions was performed using the Kaplan–Meier method and differences were compared using the log-rank test. A *P*-value of <0.05 (two-sided) was considered significant. EFS and overall survival (OS) were defined as the times from diagnosis to event (any death, relapse, secondary malignancy, and failure to therapy) and from diagnosis to death from any cause or the last follow up. Patients without an event of interest were censored at the date of last contact. The median follow-up time for EFS and OS was 5.2 years. Hazard ratios for probability of relapse between subgroups were calculated using univariate Cox models. Other comparisons were performed using the *χ*^2^ test, Fisher's exact test, and Mann–Whitney *U*-test, as appropriate.

## Results

### Patient characteristics and basic cytogenetic data in JACLS cohort

The fusion transcript of *TCF3-PBX1* was detected in 82 (7.2%) of the 1138 patients in the JACLS cohort. The characteristics of these 82 patients are summarized in Table 1 and 2. The median age at diagnosis was 6 years (range, 1−15 years), and 38 were males and 44 were females. The median leukocyte count at diagnosis was 21,750 × 10^9^/L (range 2,700−183,300). Forty-nine patients were classified as NCI-SR and 33 patients as NCI-HR. Seventy-three patients were included in the prednisolone good responder (PGR) group and nine patients in the prednisolone poor responder (PPR) group. The translocation was balanced in 17 (20.1%) cases and unbalanced in 25 (30%) cases. Either balanced or unbalanced translocation was not determined in 17 cases, including seven cases with karyotypic failure. Twenty-three (28.1%) cases with normal karyotype and seven (8.5%) cases with karyotypic failure were identified as *TCF3-PBX1* positive by RT-PCR. Fifty-three (64.6%) of the 82 patients were selected for further genetic analysis on the basis of the availability of material for testing. A comparison of the clinical characteristics of patients with and without available DNA/RNA specimens is shown in Table 1. No major differences were observed between the analyzed and nonanalyzed cohorts except for initial WBC count and NCI risk group.

**Table 1:** Comparison of the characteristics of 112 BCP-ALL patients with *TCF3-PBX1* fusion between those included and not included in the genetic analyses

Study protocol	JACLS ALL02			CCLSG ALL2004		
		
Number of patients	53	29	*P*-value	22	8	
	Analyzed	Nonanalyzed		Analyzed	Nonanalyzed	*P*-value
Sex (male/female)	28/25	10/19	0.16	9/13	3/5	1.0
Age (years) at diagnosis, median (range)	5 (1−14)	6 (1−15)	0.27	7 (1−14)	9 (2−14)	0.57
WBC count, cells/*μ*L median (range)	24,500 (4700−183,300)	16,900 (4700−55,220)	<0.01	17,100 (3200−118,000)	27,800 (9000−137,360)	0.26
NCI risk group, SR/HR	27/26	22/7	0.04	10/12	3/5	1.0
Chromosome			0.23			0.90
Normal karyotype	13	10		3	1	
t(1;19)(q23;p13)	11	6		6	3	
der t(1;19)(q23;p13)	20	5		12	4	
Unknown	9	8		1	0	
SCT in 1st CR (*n*)	2	0		ND	ND	
Observation period, median (range)	5.7 (1.6−8.7)	4.7 (0.1−8.8)	0.47	6.4 (2.3−7.7)	4.5 (3.5−7.4)	0.02
Relapse, *n* (%)	8 (15.1)	1 (3.4)	0.15	4 (18.2)	2 (25.0)	0.65
Survival			1.0			1.0
Alive, *n* (%)	45 (84.9)	25 (86.2)		20 (90.1)	7 (87.5)	
Dead, *n* (%)	8 (15.1)	4 (13.8)		2 (9.9)	1 (12.5)	

JACLS, Japan Association of Childhood Leukemia Study; CCLSG, Children's Cancer and Leukemia Study Group; WBC, white blood cell; NCI, National Cancer Institute; SR, standard risk; HR, high risk. SCT, stem cell transplantation; CR, complete remission; ND, not determined.

**Table 2:** Summary of the overall results of clinical trials of pediatric BCP-ALL with *TCF3-PBX1* from major research groups

	*n*	Sex (male/female)	Age (years) at diagnosis, median (range)	WBC count, cells/*μ*L, median (range)	CNS relapse (%)	EFS (y)	OS (y)	Reference
I-BFM (I-ALL90, 96, 12-ALLIC02)	48	22/26	ND (0.8−16)	ND (1400−434,000)	0	85	ND	6
SJCRH XIIIa-XV	41	18/23	ND	ND	9	84 (5)	96.4 (5)	5
MRC-ALL97/99	50	ND	ND	ND	6	80 (5)	84 (5)	2
NOPHO-ALL1992 and 2000	47	21/26	7 (1−18)	16,000 (1300−159,000)	0	79 (5)	85 (5)	4
CCLSG ALL2004	30	12/18	7 (1−14)	17,550 (3200−137,360)	6.7	82.8 (5)	86.3 (5)	Present study
JACLS ALL02	82	38/44	6 (1−15)	21,750 (2700−183,300)	1.2	85.4 (3)	89.0 (3)	Present study

CNS, central nervous system; EFS, event-free survival; OS, overall survival; I-BFM, International Berlin-Frankfurt-Munster; SJCRH, St. Jude Children's Research Hospital; MRC, Medical Research Council; NOPHO, Nordic Society of Pediatric Hematology Oncology; JACLS, Japan Association of Childhood Leukemia Study; CCLSG, Children's Cancer and Leukemia Study Group ND, not described.

### Patient characteristics and basic cytogenetic data in CCLSG cohort

The fusion transcript of *TCF3-PBX1* was detected in 30 (11.4%) of the 264 patients in the CCLSG cohort. The characteristics of these 30 patients are also summarized in Table 1 and 2. The median age at diagnosis was 7 years (range, 1−14 years), and 12 were males and 18 were females. The median leukocyte count at diagnosis was 17,550 × 10^9^/L (range 3200−137,360). Thirteen patients were classified as NCI-SR and 17 patients as NCI-HR. All evaluable 27 patients were included in the PGR group. The translocation was balanced in 9 (30%) cases and unbalanced in 16 (53.3%) cases. Four (13.3%) cases with normal karyotype and 1 (0.03%) case with karyotypic failure were identified as *TCF3-PBX1* positive by RT-PCR. Twenty-two (73.3%) of the 30 patients were selected for further genetic analysis on the basis of the availability of material for testing. A comparison of the clinical characteristics of patients with and without available DNA/RNA specimens is shown in Table 1. No major differences were observed between the analyzed and nonanalyzed cohorts.

### Conventional adverse prognostic factors were not associated with poor OS in the patients with *TCF3-PBX1* fusion

**Figure 1:**
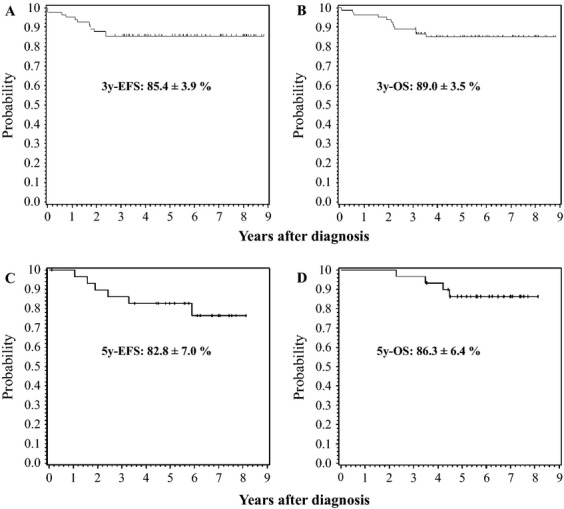
Probability of event-free survival (EFS) and overall survival (OS) in pediatric B-cell precursor acute lymphoblastic leukemia patients with *TCF3-PBX1* fusion treated according to the JACLS ALL02 (*n* = 82, A and B) and CCLSG ALL2004 protocol (*n* = 30, C and D). (A and C) EFS, (B and D) OS. JACLS, Japan Association Childhood Leukemia Study; CCLSG, Children's Cancer and Leukemia Study Group.

The predicted 3-year EFS and OS rates in the 82 *TCF3-PBX1* positive patients were 85.4 ± 3.9% and 89.0 ± 3.5% in JACLS cohort, and the 5-yerar EFS and OS were 82.8 ± 7.0% and 86.3 ± 6.4% in CCLSG cohort, respectively, which were comparable to the rates reported in western countries (Fig. 1 and Table 2). In JACLS cohort, nine (11%) of the 82 patients experienced relapse. The site of relapse was the BM in eight patients and the central nervous system (CNS) in one patient. Eight of nine patients experienced relapse during maintenance therapy, and one after allogeneic stem cell transplantation (allo-SCT) during the first CR. Four of the nine patients died of disease progression. The remaining five patients received allo-SCT. However, two of them died of transplant-related complications, and the remaining three eventually experienced relapse and died of disease progression. A comparison of the characteristics of patients according to the occurrence of relapse is shown in Table 3. The results of univariate analysis showed that none of the conventional prognostic factors, including age at onset, initial WBC count, NCI risk group, and BM status on days 15 and 33, were associated with poor EFS/OS in the 82 patients (Table S2). Although data on MRD on days 15 and 33 were not available for all patients, no statistically significant differences in MRD on days 15 and 33 were detected between nonrelapsed and relapsed patients (Figure S1).

**Table 3:** Comparison of the characteristics of BCP-ALL patients with *TCF3-PBX1* fusion according to relapse status in JACLS ALL02 and CCLSG ALL 2004 cohorts

	JACLS ALL02			CCLSG ALL2004		
		
	Relapsed	Nonrelapsed	*P*-value	Relapsed	Nonrelapsed	*P*-value
Number of patients	9	73		5	25	
Gender (male/female)	3/6	35/38	0.49	1/4	11/14	0.32
Age (years) at diagnosis, median (range)	7 (1−14)	5 (1−15)	0.81	11 (4–14)	6 (1–14)	0.05
WBC count, cells/*μ*L median (range)	24,500 (9700−70,400)	21,750 (2700−183,300)	0.08	14,500 (9400–26,600)	17,100 (3200–137,360)	0.88
NCI risk group, SR/HR	4/5	45/28	0.53	1/4	12/13	0.25
Chromosome			0.12			0.95
Normal karyotype	4	19		1	3	
t(1;19)(q23;p13)	1	16		2	7	
der t(1;19)(q23;p13)	2	23		1	12	
Unknown	2	15		1	3	
SCT in 1st CR (*n*)	1	1	0.21	ND	ND	
Survival			<0.01			<0.01
Alive, *n* (%)	0 (0)	70 (95.6)		1 (25)	25 (100)	
Dead, *n* (%)	9 (100)	3 (4.4)		4 (75)	0 (0)	

JACLS, Japan Association of Childhood Leukemia Study; CCLSG, Children's Cancer and Leukemia Study Group; WBC, white blood cell; NCI, National Cancer Institute; SR, standard risk; HR, high risk. SCT, stem cell transplantation; CR, complete remission; ND, not determined.

In CCLSG cohort, five (16.7%) of the 30 patients experienced relapse. The site of relapse was the BM in four patients and the CNS combined with BM in one patient. Three of five patients experienced relapse during maintenance therapy. Four of the five patients eventually died. A comparison of the characteristics of patients according to the occurrence of relapse is shown in Table 3. The results of univariate analysis also showed that none of the conventional prognostic factors, including age at onset, initial WBC count, and NCI risk group were associated with poor OS in the 30 patients (Table S2).

### *IKZF1* deletion is identified in relapsed BCP-ALL patients with *TCF3-PBX1* in the JACLS ALL02 cohort

The results of the MLPA analysis were summarized in Tables 4 and 5. Deletion of the *IKZF1* gene was detected in 2 (3.8%) of 53 patients with available DNA sample of diagnostic leukemic blasts, which was a significantly lower frequency than that of pediatric BCP-ALL patients without *TCF3-PBX1* fusion (3.8% vs. 10.4%, *P* < 0.001, Table 4). The *JAK2* mutation was not identified in the two patients with *IKZF1* deletion. Deletions of *PAX5*, *CDKN2A*, and *CDKN2B* were also less frequent in BCP-ALL patients with than in those without *TCF3-PBX1* fusion. However, deletion of *RB1* was detected in nine (17.0%) of 53 patients, which was a higher rate than that of patients without *TCF3-PBX1* fusion (3.0%) (Table 4). In terms of the prognostic impact of these micro deletions, none were associated with an increase of relapse except the *IKZF1* deletion (Table 5).

**Table 4:** Summary of the results of MLPA analyses of *TCF3-PBX1* positive and negative BCP-ALL patients in the JACLS ALL02 and CCLSG cohorts

	JACLS ALL02 cohort			CCLSG cohort		
		
	*TCF3-PBX1* (+)	*TCF3-PBX1* (−)	*P*-value	*TCF3-PBX1* (+)	*TCF3-PBX1* (−)	*P*-value
Number of patients	53	163		22	155	
*IKZF1* deletion (%)	2 (3.8)	17 (10.4)	<0.001	1 (4.5)	21 (13.5)	0.23
*CDKN2A* deletion (%)	10 (18.9)	71 (43.6)	<0.001	6 (27.3)	37 (23.9)	0.73
*CDKN2B* deletion (%)	8 (15.1)	61 (37.4)	<0.001	5 (22.7)	40 (25.8)	0.76
*PAX5* deletion (%)	12 (22.6)	47 (28.8)	0.37	9 (40.9)	28 (18.1)	0.014
*ETV6* deletion (%)	1 (1.9)	46 (28.2)	<0.001	2 (9.1)	40 (25.8)	0.085
*RB1* deletion (%)	9 (17.0)	3 (1.8)	<0.001	4 (18.2)	20 (12.9)	0.50
*BTG1* deletion (%)	1 (1.9)	14 (8.6)	<0.001	1 (4.5)	20 (12.9)	0.26
*EBF1* deletion (%)	2 (3.8)	20 (12.3)	<0.001	2 (9.1)	17 (10.9)	0.79

JACLS, Japan Association of Childhood Leukemia Study; CCLSG, Children's Cancer and Leukemia Study Group.

**Table 5:** Impact of genetic alterations on the outcomes of patients with BCP-ALL with *TCF3-PBX1*

	JACLS ALL02 cohort			CCLSG cohort		
		
	Relapse	Nonrelapse	*P*-value	Relapse	Nonrelapse	*P*-value
Number of patients	8	45		4	18	
*IKZF1* deletion (%)	2 (25)	0	<0.01	1 (25)	0	0.03
*CDKN2A* deletion (%)	1 (12.5)	9 (20)	0.62	2 (50)	4 (22.2)	0.26
*CDKN2B* deletion (%)	1 (12.5)	7 (15.6)	0.99	2 (50)	3 (16.7)	0.15
*PAX5* deletion (%)	2 (25)	10 (22.2)	0.72	2 (50)	7 (38.9)	0.68
*ETV6* deletion (%)	0	1 (2.2)	0.67	0	2 (11.1)	0.48
*RB1* deletion (%)	2 (25)	7 (15.6)	0.51	0	4 (22.2)	0.30
*BTG1* deletion (%)	0	1 (12.5)	0.67	1 (12.5)	0	0.18
*EBF1* deletion (%)	0	2 (25)	0.54	0	2 (11.1)	0.48

JACLS, Japan Association of Childhood Leukemia Study; CCLSG, Children's Cancer and Leukemia Study Group.

*TP53* mutation has been associated with relapse in BCP-ALL patients with *TCF3-PBX1* fusion 8. Therefore, we screened for mutations in *TP53* in the diagnostic samples of eight cases experiencing relapse. In addition, *TP53* mutation screening was performed in relapsed leukemic samples from four of the eight relapsed patients. However, *TP53* mutations were not observed in these 12 samples.

### *IKZF1* deletion is also identified in relapsed patient with *TCF3-PBX1* positive BCP-ALL in the validation cohort (CCLSG cohort)

To confirm the significance of the *IKZF1* deletion in BCP-ALL with *TCF3-PBX1*, 22 diagnostic leukemic samples with *TCF3-PBX1* fusion obtained from the patients registered in the CCLSG ALL2004 protocol were analyzed by MLPA. Although only 1 (4.5%) of 22 patients had the *IKZF1* deletion, this patient experienced relapse and died of the disease (Tables 4 and 5).

## Discussion

In this study, we showed that *TCF3-PBX1* positive pediatric BCP-ALL patients treated according to the JACLS ALL02 and CCLSG ALL2004 protocol had favorable outcomes similar to those reported in western countries (Table 2) 2,4–6. A low frequency of CNS relapse (1 in 82 patients, 1.2%) was observed in JACLS ALL02 cohort, showing a more favorable result than those reported in the St. Jude and MRC cohorts (Table 2) 2,5. However, 9 (11%) of 82 in JACLS and 5 (16.7%) of 30 patients in CCLSG cohort experienced relapse and most of them died of disease progression or transplant-related complications. The clinical and biological features of the relapsed cases should be evaluated to further improve the prognosis of pediatric BCP-ALL with *TCF3-PBX1* fusion. To the best of our knowledge, a reliable prognostic marker has not been identified in this subgroup 4,5,7. In this study, conventional prognostic markers, such as age at onset, initial WBC count, and NCI risk group, were not associated with poor prognosis, which was in agreement with previous studies. Furthermore, we found that the type of chromosomal abnormality had no prognostic impact 4,5. However, the pattern of relapse was unique in the present study, especially in JACLS cohort: eight of nine patients relapsed during the maintenance phase and one patient had primary induction failure, suggesting that leukemic blasts in these patients initially showed resistance to the chemotherapeutic agents used. However, BM status and assessment of MRD on days 15 and 33 did not identify those patients who experienced very early relapse, suggesting that a small fraction of leukemic cells contributed to relapse.

Few studies have investigated the association between genetic alterations and prognosis in BCP-ALL with *TCF3-PBX1*. Kawamura et al. identified point mutations of *TP53* in two of 20 initial patients and in four relapsed patients with BCP-ALL expressing *TCF3-PBX1* and suggested a possible relationship between *TP53* mutation and disease progression in BCP-ALL patients with *TCF3-PBX1* fusion 8. Therefore, in this study, we screened for *TP53* mutations in *TCF3-PBX1* positive BCP-ALL patients who experienced relapse. However, no mutations in *TP53* (exons 5−9) were detected in four relapsed leukemia samples of eight relapsed patients. Furthermore, *TP53* mutation analysis of the diagnostic samples of eight relapsed patients did not show any mutations. However, because the prognosis of BCP-ALL with *TCF3-PBX1* fusion has improved dramatically in the last decades, *TP53* mutations might not be associated with relapse in this subgroup.

*IKZF1* deletion is known to be a strong prognostic factor associated with poor outcome in pediatric BCP-ALL 11,14–16. However, the significance of *IKZF1* deletion in BCP-ALL with *TCF3-PBX1* fusion has not been determined. Previous studies have shown that *IKZF1* deletion is less frequent in BCP-ALL with recurrent chromosomal abnormalities 11,14–16, which is in agreement with our results showing that the frequency of *IKZF1* deletion was lower in the *TCF3-PBX1* fusion positive subgroup than in the other subgroups (Table 4). However, *IKZF1* deletion was detected in 2 (25%) of eight evaluable relapsed patients, whereas it was not present in any of the patients maintaining continuous CR (Table 5). In addition, MLPA analysis of 22 diagnostic samples of BCP-ALL with *TCF3-PBX1* in the independent CCLSG cohort identified one patient with *IKZF1* deletion who eventually relapsed and died. Although our observation may be potentially interesting, further study employing larger cohort is warranted to determine the prognostic impact of *IKZF1* deletion in this subgroup.

Ferreiros-Vidal et al. reported that Ikaros-regulated genes are highly represented in pre-Bcell receptor signaling, cell cycle regulation, and the somatic rearrangement of Ig genes, which are key to the differentiation of B-cell progenitors 17. These authors showed that inducible Ikaros expression in cycling pre-B cells was sufficient to drive transcriptional changes, such as repression of *Myc*, *Cdk6*, *Ccnd2*, *Cdkn1a*, and *Cdkn1b*, resembling the differentiation of cycling to resting pre-B cells in vivo. These findings suggested that haplo-insufficiency of *IKZF1* might be associated with the differentiation block and accelerated cell cycle progression in BCP-ALL. In that study, 52% of TCF3 target genes were also bound by Ikaros, suggesting that these two transcriptional factors collaborate to regulate B-cell specification. These findings indicate that a severe impairment in the differentiation of cycling to resting pre-B cells in BCP-ALL with *IKZF1* deletion and *TCF3-PBX1* fusion may be associated with poor prognosis.

In this study, no additional genetic alterations were detected in ∼75% of relapsed patients with *TCF3-PBX1*. To identify additional genetic alterations related to poor prognosis, a comprehensive genomic analysis using matched pair samples of onset and relapse might be useful. Our group is therefore planning to perform exome analysis of relapsed and nonrelapsed patients with BCP-ALL expressing *TCF3-PBX1*.

In conclusion, the prognosis of pediatric BCP-ALL patients with *TCF3-PBX1* fusion treated according to the JACLS ALL02 and CCLSG ALL2004 protocol was similar to that reported in studies performed in Western countries. Although concomitant *IKZF1* deletion may account for ∼25% of treatment failure in this subgroup, further study of larger cohort is warranted to determine the prognostic impact of *IKZF1* deletion in this subgroup.

## Supporting Information

**Table S1**. The primer list of *TCF3-PBX1* and *TP53* used in the current study.

**Table S2**. Univariate Cox model of event-free and overall survival of 112 patients with *TCF3-PBX1*

**Figure S1**. Comparison of minimal residual disease (MRD) between relapsed and nonrelapsed patients determined by quantitative RT-PCR of the *TCF3-PBX1* transcript on days 15 and 33 of the induction phase in JACLS cohort: (A) day 15 and (B) day 33.
