# Supplementary figures and images for: Outcome of TCF3-PBX1 positive pediatric acute lymphoblastic leukemia patients in Japan: a collaborative study of Japan Association of Childhood Leukemia Study (JACLS) and Children's Cancer and Leukemia Study Group (CCLSG)
Source: Cancer Med. 2014 Feb 28;3(3):623–31. doi: 10.1002/cam4.221 (PMC4101753; doi:10.1002/cam4.221)

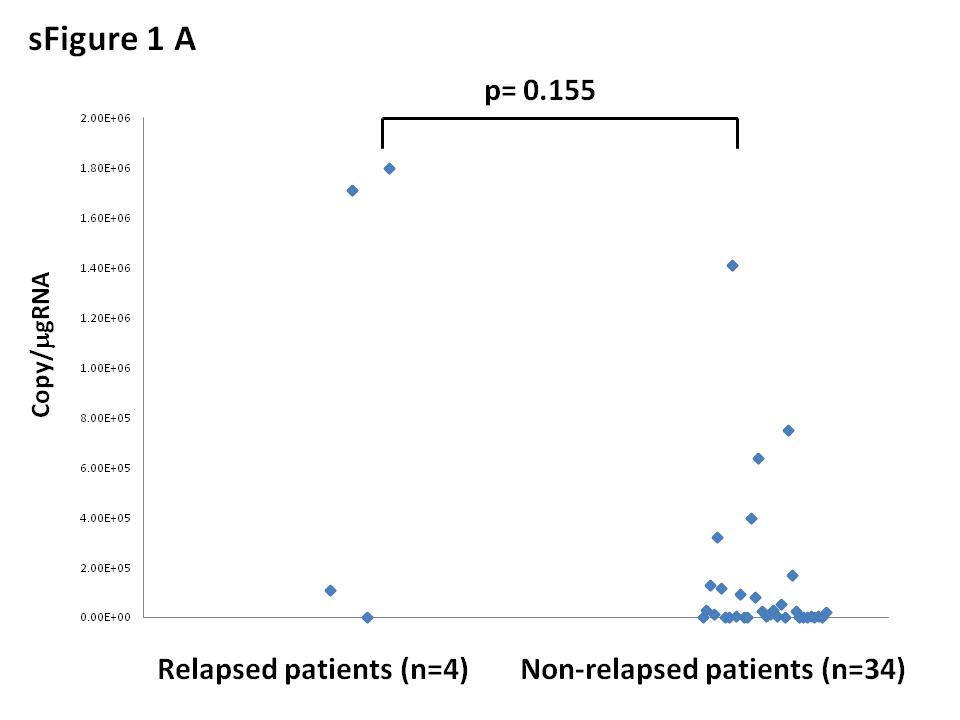

Supplement: Supplementary file 1 — Table S1. The primer list of TCF3-PBX1 and TP53 used in the current study. [file cam40003-0623-SD1.tif]

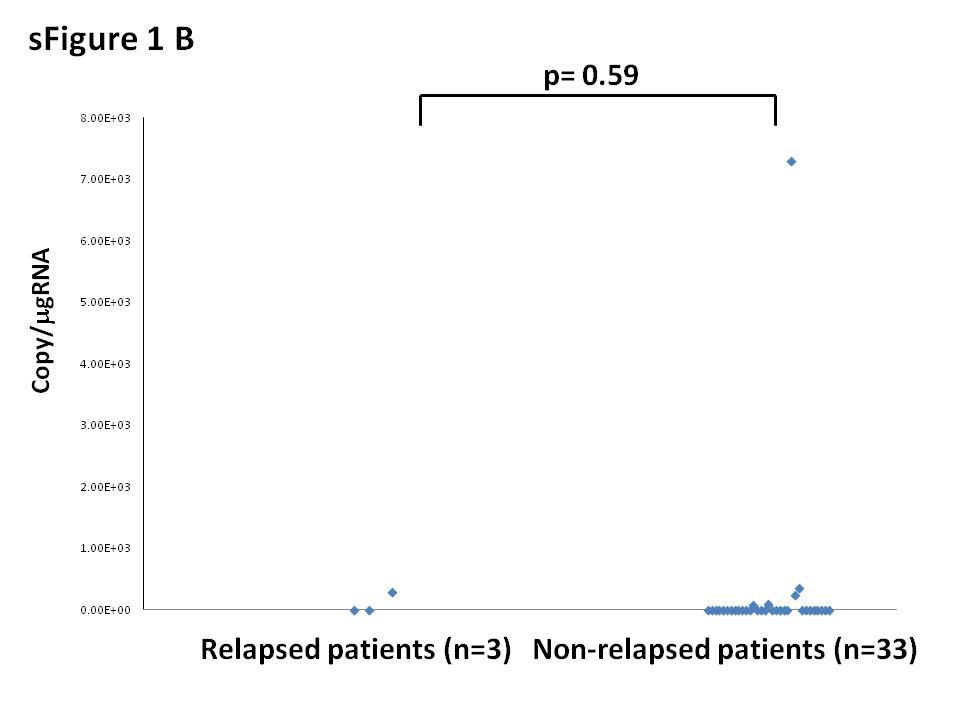

Supplement: Supplementary file 2 — Table S2. Univariate Cox model of event-free and overall survival of 112 patients with TCF3-PBX1 [file cam40003-0623-SD2.tif]
