# Supplementary material for: Outcome of TCF3-PBX1 positive pediatric acute lymphoblastic leukemia patients in Japan: a collaborative study of Japan Association of Childhood Leukemia Study (JACLS) and Children's Cancer and Leukemia Study Group (CCLSG)
Source: Cancer Med. 2014 Feb 28;3(3):623–31. doi: 10.1002/cam4.221 (PMC4101753; doi:10.1002/cam4.221)
Supplement: Supplementary file 3 — Figure S1. Comparison of minimal residual disease (MRD) between relapsed and nonrelapsed patients determined by quantitative RT-PCR of the TCF3-PBX1 transcript on days 15 and 33 of the induction phase in JACLS cohort: (A) day 15 and (B) day 33. [file cam40003-0623-SD3.docx]

**Supplementary Table 1**: The list of primer sets for *TCF3-PBX1* fusion and *TP53* used in the current study

| *TCF3-PBX1* |  | Forward | CAG CAC CAG CCT CAT GCA CA |
| --- | --- | --- | --- |
|  |  | Reverse | GTT GTC CAG CCG CAT CAG CT |
|  |  | Probe | FAM-TTG AGT ATC CGA GGA GCC CAG GAG GAG GAA-TAMRA |
| *TP53* | Exon 5-6 | Forward | GCCCTGACTTTCAACTCTGTCTC |
|  |  | Reverse | GGCCACTGACAACCACCCTTAACC |
|  | Exon 7 | Forward | AAAGGCCTCCCCTGCTTGCC |
|  |  | Reverse | CCGGGGATGTGATGAGAGGTGG |
|  | Exon 8-9 | Forward | ACCTGATTTCCTTACTGCCTCTTGC |
|  |  | Reverse | TGGCAAATGCCCCAATTGCAGG |

**Supplementary Table 2**: Univariate Cox model of event-free and overall survival of patients with *TCF3-PBX1*

|  | JACLS ALL02 | | | CCLSG ALL2004 | | |
| --- | --- | --- | --- | --- | --- | --- |
| Variable | Hazard ratio | p | 95% CI | Hazard ratio | p | 95% CI |
| **Event-free surviva**l |  |  |  |  |  |  |
| Age (yrs) at diagnosis (10−18 vs.1-9) | 2.130 | 0.20 | 0.676−6.712 | 6.909 | 0.047 | 0.879-54.300 |
| Sex (male vs. female) | 0.844 | 0.77 | 0.268−2.661 | 0.375 | 0.62 | 0.047-2.960 |
| NCI risk (HR vs. SR) | 1.471 | 0.50 | 0.474−4.561 | 3.059 | 0.35 | 0.386-24.216 |
| Day 15 BM status (M2+M3 vs. M1) | 1.888 | 0.34 | 0.507−7.032 | ND |  |  |
| **Overall survival** |  |  |  |  |  |  |
| Age (yrs) at diagnosis (10−18 vs.1-9) | 2.100 | 0.28 | 0.666−6.616 | 5.182 | 0.12 | 0.610-43.953 |
| Sex (male vs. female) | 0.828 | 0.74 | 0.263−2.609 | 0.500 | 0.63 | 0.059-4.259 |
| NCI risk (HR vs. SR) | 1.440 | 0.53 | 0.464−4.465 | 2.294 | 0.61 | 0.269-19.594 |
| Day 15 BM status (M2+M3 vs. M1)* | 1.938 | 0.32 | 0.520−7.218 | ND |  |  |

*ALL 82 patients showed M1 marrow at day 33 post induction therapy. M1, M2, and M3 marrow was defined as fewer than 5%, 5 to 25%, and more than 25% blast cells in the bone marrow aspirate, respectively.

Abbreviations: JACLS, Japan Association of Childhood Leukemia Study; CCLSG, Children’s Cancer and Leukemia Study Group; NCI, National Cancer Institute; SR, standard risk; HR, high risk. BM, bone marrow.
